# Bioimaging tools move plant physiology studies forward

**DOI:** 10.3389/fpls.2022.976627

**Published:** 2022-09-20

**Authors:** An-Shan Hsiao, Ji-Ying Huang

**Affiliations:** ^1^ Biosciences, College of Life and Environmental Sciences, University of Exeter, Exeter, United Kingdom; ^2^ Cell Biology Core Lab, Institute of Plant and Microbial Biology, Academia Sinica, Taipei, Taiwan

**Keywords:** Cell Biology, plant physiology, Microscopy, biosensor, microfluid, chemicals

## Introduction

**Figure 1:**
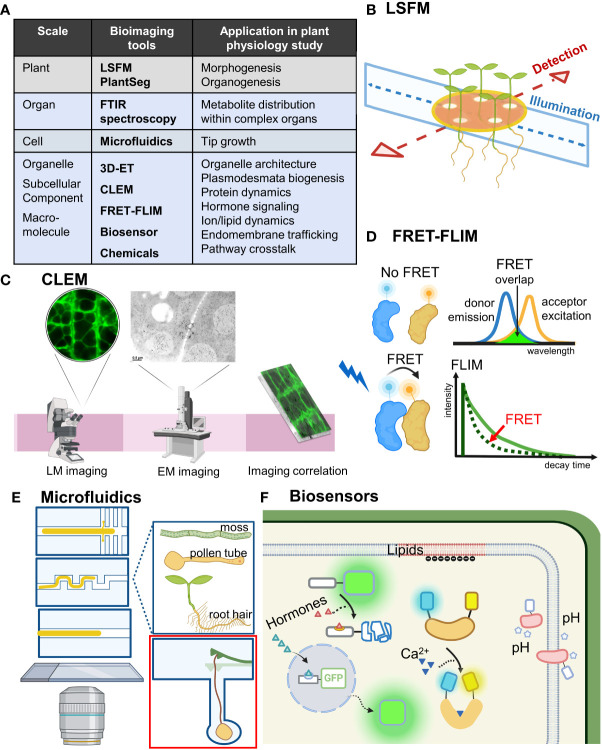
Bioimaging tools move plant physiology studies forward. **(A)** Bioimaging tools ranged from an organism scale to a subcellular scale. Their application in plant physiology research is shown. Bioimaging tools mentioned in the text are in bold. **(B)** LSFM achieves optical sectioning by selective illumination on one single plane with a sheet of laser light while simultaneously detecting emitted fluorescence orthogonal to the illumination plane. Incorporating MAGIC, LSFM can allow for high-throughput time-course imaging of multiple samples under near-physiological conditions. **(C)** Combining the advantages of electron and light microscopy-based imaging, CLEM enables precise molecular localization and provides structure–function analysis. **(D)** By measuring the lifetime of the FRET donor fluorescence, FRET-FLIM can detect location-specific protein–protein interaction *in vivo*. **(E)** Microfluid techniques can be flexibly designed and adjusted and are especially useful for observing tip growth cells such as root hairs, pollen tubes and moss. Red inset shows a microfluidic lab-on-a-chip device for quantifying the invasive growth force of pollen tubes developed by Ghanbari et al. **(F)** Biosensors for detecting hormones, Ca^2+^, pH and lipids allow for real-time studies of cell physiology and signaling events with high spatial and temporal resolution. LSFM, light sheet fluorescence microscopy; FTIR, Fourier-transform infrared; 3D-ET, three-dimensional electron tomography; CLEM, correlative light and electron microscopy; FRET-FLIM, Förster resonance energy transfer-fluorescence lifetime imaging microscopy; MAGIC, Multi-sample Arabidopsis Growth and Imaging Chamber.

Cell biology investigation is important for plant physiology research in terms of dissecting biological processes from plants/organs to macromolecular scales in various spatial and temporal manners and integrating these processes into plant developmental programs and stress responses. Bioimaging tools can be simply divided into two categories: “hard” microscopy and “soft” biosensors/probes. New imaging techniques for microscopy improvement have increased the speed and depth of acquisition, sensitivity, and spatial resolution (Grossmann et al., 2018; Clark et al., 2020). The development of improved fluorescent proteins, genetically encoded biosensors/reporters and pharmaceutical treatments have helped in measuring the spatiotemporal dynamics of cell physiological parameters (Uslu and Grossmann, 2016; Rodriguez-Furlan et al., 2017; Colin et al., 2022). Readers are invited to visit the excellent review papers on microscopy techniques and probe development (Uslu and Grossmann, 2016; Grossmann et al., 2018; Clark et al., 2020; Colin et al., 2022). Because of the importance of bioimaging tools for advancing study of mechanisms in plant physiology research, here we discuss combinatory microscopy improvement and biosensor innovation, describing their application from morphogenesis to macromolecule dynamics. We discuss the microdevice innovation in tip growth study and the latest chemical cell biology application, which provide reversible and conditional approaches to dissect the intracellular dynamics overcoming the problems of redundancy and lethality in plant physiology study (**Figure 1A**). For the Research Topic “Women in Plant Physiology”, this article emphasizes the intelligence and innovation of female scientists in the recent progress of bioimaging techniques. By highlighting their contributions and efforts to move the field forward, we aim to encourage the younger generation of female scientists interested in the study of cell biology and plant physiology.

## Combinatory microscopy improvement: From morphogenesis to protein dynamics

Light sheet fluorescence microscopy (LSFM) has been advanced to achieve long-term live imaging by significantly reducing phototoxicity with fast acquisition of 3D data over time. The upright sample position in most LSFM setups is well suited for studying the fundamental aspects of plant organogenesis (Berthet and Maizel, 2016). However, certain challenges remain, such as the ability to image only one specimen at a time and the stress response generated by the imaging capillary system. Rosangela Sozzani and her team developed a Multi-sample Arabidopsis Growth and Imaging Chamber (MAGIC) that provides near-physiological imaging conditions and allows for high-throughput time-course imaging experiments with the ZEISS Lightsheet Z.1 microscope, with a semi-automatic image processing pipeline for data analysis (de Luis Balaguer et al., 2016; **Figure 1B**). Their innovation scales up the existing commercial platforms with up to 48-h imaging capacity and efficient multiplexing of up to 12 seedlings, which can be used for imaging developmental processes such as root growth, cell division events as well as light-controlled experiments.

Microscopy improvement such as LSFM allows for capturing the anatomy and development of plants in terabytes of high-resolution volumetric images. However, accurate segmentation of individual cells in volumetric images of growing organs is needed. Anna Kreshuk and her colleagues released PlantSeg, an open-source software for 2D and 3D segmentation of cells with cell contour staining (Wolny et al., 2020). Recent research combining PlantSeg and ClearSee-based staining methods revealed a cellular growth pattern during Arabidopsis ovule development (Vijayan et al., 2021). Hence, PlantSeg can be used with modern clearing methods to study various developmental events of diverse plant organs and even animal tissues.

Time-course live imaging experiments are critical for understanding dynamic cellular processes. When cells undergoing cell division grow in length, studying them requires manual adjustment of the observation field over time. Thus, Marie-Cécile Caillaud and her colleagues developed a protocol for automatic time-lapse imaging of multiple growing root tips for several hours, which allows for easy temporal observation of cytokinesis and other cellular processes (Doumane et al., 2017).

Ljudmilla Borisjuk and her team established an imaging platform based on Fourier-transform infrared spectroscopy for analysing cereal crops (Gündel et al., 2018). This platform allows for the quantitative visualization of sucrose in individual vascular bundles or complex organs with high sensitivity and resolution. It can resolve the spatial distribution of metabolites and carbon allocation as well as storage in the context of crop improvement (Gündel et al., 2018).

Understanding 3D architectures of organelles during various developmental stages in different cell types is important for analyzing their functions in plant physiology research. Electron tomography (ET) approaches can retrieve 3D structural information from a series of 2D projections of a biological specimen at different angles to provide 3D structural evidence (Ercius et al., 2015; Otegui and Pennington, 2019). Marisa Otegui and her colleagues established ET approaches to analyze vesicular trafficking and *de novo* assembly of plant cell walls (Otegui and Pennington, 2019; Otegui, 2020). Their recent findings show that endosomal intralumenal vesicles are formed by a concatenation process in Arabidopsis root and tapetal cells (Buono et al., 2017; Goodman et al., 2021). This process contrasts with the current model established in the mammalian system in which endosomal intralumenal vesicles are free and formed individually (Murk et al., 2003). Therefore, the establishment of bioimaging tools in plants is critical to investigate the novel mechanism in plant cells, which are distinct from mammalian cells.

Combining electron microscopy and light microscopy, correlative light and electron microscopy (CLEM) can be used to visualize the ultrastructure for studying intracellular function (Begemann and Galic, 2016; **Figure 1C**). Lysiane Brocard and her colleagues developed a CLEM approach to study the graft interface of Arabidopsis grafting hypocotyls (Chambaud et al., 2022). With in-resin fluorescence CLEM combined with electron tomography, this method can provide the fine 3D ultrastructural details with up to the resolution of the bilayer of the plasma membrane (Chambaud et al., 2022). Classical CLEM usually results in low-resolution correlations because it is limited to the resolution of the light microscope, but this method can detect fluorescence signals and more accurately determine the ultrastructure position within electron tomograms.

Förster resonance energy transfer (FRET) is a well-established technique to study molecular interactions by monitoring non-radiative energy transfer from an excited fluorescent donor to a non-excited different fluorescent acceptor (Förster, 1948; Lakowicz, 2006; Müller et al., 2013). Fluorescence lifetime imaging microscopy (FLIM) can deliver information about the spatial distribution of a fluorescent molecule with its biochemical status or nano-environment (Brismar and Ulfhake, 1997; van Munster and Gadella, 2005). Determining the lifetime of the FRET donor fluorophore by FLIM, FRET-FLIM has been widely used to detect and visualize protein interactions spatially (Fäßler and Pimpl, 2017; Weidtkamp-Peters and Stahl, 2017; **Figure 1D**). Ikram Blilou and her team optimized FRET-FLIM technology in living Arabidopsis roots to show the physical proximity between the C2H2-type transcription factor JACKDAW, the mobile protein and cell fate regulator SHORTROOT, and its target SCARECROW compartments (Long et al., 2017). This optimized FRET-FLIM technology provides visualization of cell-specific protein–protein interactions, which is useful for observing cell type-specific complexes in many biological processes such as gene expression, signaling, cell size regulation, and growth.

Fluorescence correlation spectroscopy (FCS) is a powerful technique to measure fluorescence fluctuations for exploring the dynamic behaviours of proteins and the organization of membranes within living cells (Bacia et al., 2006; Li et al., 2016). Scanning FCS extends the application of FCS to receptor–ligand interactions by repeatedly scanning the detection volume through a vertical membrane perpendicularly (Ries et al., 2009). Rosangela Sozzani and her team used scanning FCS to track the mobility and interactions of the transcription factors SHORTROOT and its downstream target SCARECROW, which control root patterning and cell fate specification in plants (Clark et al., 2016). By combining scanning FCS and pair correlation functions, the team dissected the directionality of transcription-factor movement in various cell types quantitatively with a high spatiotemporal resolution. With its high fluorescence sensitivity, this FCS approach is more useful for resolving the dynamics and interactions of fast diffusing macromolecules rather than immobile molecular interactions such as stable transcription factor binding events.

As FCS is used for *in vivo* imaging of transcription factor movement and interactions (Clark et al., 2016), single-particle tracking (SPT) is a valuable analytical method for unravelling the dynamics of membrane proteins in regulating signal transduction with super-spatiotemporal resolution (Cui et al., 2018; Wang et al., 2018). Xiaojun Li and her colleagues developed a SPT method for studying the distribution and dynamics of the plant membrane protein aquaporin (Li et al., 2011; Cui et al., 2021). This method can be used to study specific membrane protein motions during abiotic and biotic stress responses by directly “observing” how particles diffuse in living cells in front of our eyes (Cui et al., 2018). This “seeing is believing” technique has great potential in investigating cell signalling and membrane rafts.

## Microfluid system for observing tip growth

Versatile microdevices have been used for live imaging of plants (Froelich et al., 2011; Kirchhelle and Moore, 2017; Clark et al., 2020). Microfluidic systems allow for live imaging of biological samples growing in defined channels with the stream of fluid continuously renewing the growth medium (**Figure 1E**). They facilitate quantitative and dynamic measurements and precisely control the microenvironment (Whitesides, 2006; Meier et al., 2010; Grossmann et al., 2011; Parashar and Pandey, 2011; Grossmann et al., 2018). Tip growth involves an expandable cell wall and localized exocytosis at the tip of a cell. Pollen tubes and root hairs are well-studied tip growth cells because of their important functions in plant breeding and nutrient uptake (de Ruijter and Malhó, 2000; Heath and Geitmann, 2000). Anja Geitmann and her team developed a multi-layer soft lithography process in a microfluidic lab-on-a-chip device featuring a microscopic cantilever to quantify the invasive growth force (Ghanbari et al., 2018; **Figure 1E** red inset). This platform can harbour growing pollen tubes while measuring instant growth force during the mechanical interactions, for a promising application for mechanobiological studies in growing cells (Ghanbari et al., 2018). The team of Anja Geitmann further improved lithography-based microfluidics as a low-cost alternative; this is a silicone-based spacer system with flexible design, which can be cleaned and reused repeatedly and allows for live cell imaging at high resolution of pollen tubes growing *in vitro* (Bertrand-Rakusová et al., 2020). Their innovation can provide a fundamental platform for studying the growth behavior and mechanosensing of pollen tubes.

With their fast apical growth, root hairs are easily visualized and accessible to variety of experimental manipulations and physiological tests, thus providing numerous advantages for basic studies of development, cell biology and physiology (Grierson et al., 2014). Marie-Edith Chabouté is interested in studying plant nuclear mechanics by using Arabidopsis root hairs as a model system. Her team invented a powerful coverslip-based microfluidic device to observe Arabidopsis root hair development with high-resolution confocal imaging as well as real-time monitoring of nuclear movement and shape changes (Singh et al., 2021). Nuclear movement is an important controlling point during development and signalling events, so their research provides a unique tool for studying the roles of nucleus dynamics in various biological processes.

The moss *Physcomitrium patens* has emerged as a good model to study tip growth in plants owing to its benefits of excellent rapid genetics and cytology (Rounds and Bezanilla, 2013; Bibeau et al., 2021). As a pioneer in moss research, Magdalena Bezanilla and her team described a continuous-culture method within microfluidic chambers for long-term imaging of development in the moss (Bascom et al., 2016). This method overcame the current long-term imaging challenges and allowed for continuous imaging over a long developmental time for weeks. These devices provide an opportunity to pursue the molecular basis of developmental events such as protonemal tissue differentiation, bud formation, and phyllid expansion at cellular and subcellular resolutions with available high-throughput pharmacological treatments.

## Biosensors for hormone signaling, dynamics of Ca^2+^, pH and lipids

Fluorescent protein-based genetically encoded biosensors are increasingly being used to visualize and analyse ion fluxes, signaling components, and metabolites for real-time studies of cellular processes with high spatial and temporal resolution (Gjetting et al., 2013; Hilleary et al., 2018; Walia et al., 2018; Walia et al., 2021; Waadt et al., 2021). Biosensors detecting hormone distribution and signaling have benefited physiology studies of plant development (Balcerowicz et al., 2021; Isoda et al., 2021; **Figure 1F**). Carolyn Rasmussen and her team modified a sensitive and dynamically responsive auxin signaling reporter based on the DII domain of Arabidopsis INDOLE-3-ACETIC ACID 28 for use in maize (Mir et al., 2017). This DII-based reporter responded to both exogenous indole-3-acetic acid and endogenous auxin, particularly transient areas of low auxin accumulation/perception, thus highlighting its utility in studying mechanisms of auxin signaling during maize development (Mir et al., 2017).

The group of Maya Bar recently reported the improved version of the cytokinin sensor two-component signaling sensor (TCS), TCSv2, with increased sensitivity and expression pattern, which is an ideal TCS version to study cytokinin response in a host plant such as tomato and tissues such as leaves and flowers (Steiner et al., 2020). Their recent research used TCSv2 to understand the balance between cytokinin and gibberellin during tomato leaf development (Israeli et al., 2021), highlighting its utility in studying organ development and shape determination as well as the relationship between cytokinin and other hormones.

Jennifer Nemhauser and her team introduced a novel set of synthetic and modular hormone-activated Cas9-based repressors (HACRs) in Arabidopsis that respond to three hormones: auxin, gibberellins and jasmonates (Khakhar et al., 2018). In the gibberellin HACRs, this approach revealed an endosperm-specific gibberellin distribution corresponding to *AtGA3ox4* expression in early seed development. Because the HACR approach is modular, other gibberellin-targeted proteins can be investigated or other pathways dependent on regulated protein degradation quantified; thus it is useful for studying hormone signalling events. A further agricultural engineering application is to use HACR technology to reprogram development by changing the hormone signalling network.

As a second messenger, Ca^2+^-mediated signaling participates in the regulation of plant cell physiology and cellular responses to the environment (Dodd et al., 2010). Melanie Krebs and Karin Schumacher established a standard protocol for combining the use of locally targeted genetically encoded calcium indicators and confocal laser scanning microscopy to measure cytosolic and nuclear Ca^2+^ dynamics in Arabidopsis roots (Krebs and Schumacher, 2013). The researchers improved the sensitivity and signal resolution of Ca^2+^ indicators from FRET-based R-GECO1 to ratiometric R-GECO1-mTurquoise (Keinath et al., 2015; Waadt et al., 2017). These Ca^2+^ indicators are useful for studying cytosolic Ca^2+^ oscillations in various cell types during fungal infection and hormonal responses related to abscisic acid and auxin, for example.

The study of pH in cellular physiology is important for understanding ionic balance, membrane regulation of ion channels and transporters as well as root growth (Moreau et al., 2021). Nadine Paris and her team generated membrane-anchored ratiometric pH sensors that allow for non-invasive pH measurement on both sides of the plasma membrane of living Arabidopsis roots (Martinière et al., 2018). By using this powerful tool, the researchers found that the cell wall plays a role in proton homeostasis in mature roots (Martinière et al., 2018). For acidic pH measurement, they further generated pH sensors with range from pH 3 to 8, called Acidins, which are useful to directly report physiological conditions related to cell elongation (Moreau et al., 2022).

Various lipid biosensors have been designed for studying lipid dynamics and membrane functions (Platre and Jaillais, 2016; Hammond et al., 2022). Marie-Cécile Caillaud and her colleagues generated a series of membrane-surface charge markers and lipid sensors to address the unique electrostatic signature of the plasma membrane, controlled by phosphatidylinositol-4-phosphate (PI4P) (Simon et al., 2016). Their recent research used a synthetic inducible system, inducible depletion of PI(4,5)P2 in plants (iDePP), to confirm that PI(4,5)P2 is critical for various aspects of plant development, including root growth, root-hair elongation and organ initiation (Doumane et al., 2021).

By using a bio-ortholog of choline, Kathrin Schrick and her team developed a click-chemistry–based method for imaging choline phospholipids in various cell types and tissues from Arabidopsis (Paper et al., 2018). Their method provides a direct way to metabolically tag and visualize specific lipid molecules in plant cells. Although click-chemistry in metabolic labelling is common in mammalian lipid research (Ancajas et al., 2020), this innovation represents a major progress in plant lipid research, which was at the stage of isotopic labeling for a long time (Allen et al., 2015). More research regarding lipid metabolism, trafficking and localization in plant cells with this approach is foreseeable.

## Chemicals for probing intracellular processes

Besides click-chemistry in biosensor application, chemicals can be used to target specific proteins to affect their functions during signal transduction or in critical pathways (Li et al., 2012; Rodriguez-Furlan et al., 2017). Therefore, they are useful to dissect molecular mechanisms regarding hormone signaling, development, cell wall biogenesis, and plant immunity as well as to investigate dynamic intracellular processes such as protein subcellular localization, secretion and trafficking (Hicks and Raikhel, 2012; Dejonghe and Russinova, 2017). Traditional chemicals with known action mechanism such as brefeldin A can be used for investigating intracellular processes influenced by plant hormones (Peyroche et al., 1999). The quantitative microscopic analysis established by Eugenia Russinova’s laboratory involves exocytosis and recycling, plasma membrane receptor dynamics and their roles in brassinosteroid signaling (Luo et al., 2015; Luo and Russinova, 2017). The team’s recent research revealed that the human and plant endomembrane system showed different responses to the chemical effector endosidin9 (Dejonghe et al., 2019). This selective inhibition effect of small molecules is useful to dissect the different roles within protein families and distinct species. Such different responses to chemicals between human and plant endomembrane systems highlighted the importance to look at them not from the perspective of animal physiology but instead to establish a plant-based chemical toolbox for physiological study (Norambuena and Tejos, 2017).

More than a decade ago, Natasha V. Raikhel foresaw the merits of using chemicals to perturb the essential physiological processes in plants to overcome the problems of redundancy and lethality. Her team has been working on the discovery and use of chemical tools to establish a chemical toolbox for plant physiological study (Hicks and Raikhel, 2012; Li et al., 2012; Hicks and Raikhel, 2014). Their research has revealed various valuable chemical probes such as Sortin1 for dissecting the regulation among flavonoid transport, vacuolar integrity and trafficking (Rosado et al., 2011) and endosidin17 for understanding the checkpoint for membrane fusion with the vacuole (Rodriguez-Furlan et al., 2019). Her foresight in using chemical tools for visualizing and manipulating cellular processes has led to interesting discoveries and prompted a new research area of chemical genetics for studying the functions of essential plant proteins.

## Always moving forward

Compared to her research, the life story of Natasha V. Raikhel is even more encouraging (Raikhel, 2017). She was born in Germany and moved to Russia, where she grew up as a budding pianist and later changed her career goal from music to biology. Although it was a difficult realization that she was not destined to become a professional pianist, her pragmatism and hard work drove her to catch up in biology and chemistry at university, where she used classical cell biology for studying ciliates. The ciliate work was boring and descriptive and the fact that she could not ask deeper biological questions led to decisions that would lay the foundation for other scientists. During the difficult time, she always faced unfairness, shortages and limitations in both research and life, and the last straw was a horrific plane crash in 1978, which led to her decision to immigrate to the United States. There, she was reborn as a plant biologist studying lectin, cell wall and vesicular trafficking as well as the aforementioned chemical genomics tools for plant physiology research. In 1998, she was diagnosed with breast cancer, but just like other difficulties she met, she overcame the disease. She continued to work in the laboratory during the chemotherapy treatments, and after her recovery, she became the first and (so far) only female editor in chief of the journal *Plant Physiology* during 2000-2005, where she made a difference by highlight the latest technology-driven insights in plant biology. She retired from her university career in 2016, but she maintained her work in promoting women in science and music all around the world. The story of Natasha V. Raikhel represents how a female scientist overcame difficulties and pushed the field forward, just like other examples of female scientists have led to significant progress in plant physiology research. Changes and challenges always happen in life and research, if you don’t know where to go, any road will lead you there. Just keep going and moving forward!

## Author contributions

A-SH developed the concept and wrote the manuscript. A-SH and J-YH produced the data in **Figure 1**. J-YH prepared **Figure 1**. The authors contributed to the article and approved the submitted version.

## Funding

A-SH was supported by a BBSRC research grant (BBSRC BB/T005424/1) led by Prof. Nicholas Smirnoff.

## Acknowledgments

Confocal and immunogold TEM images were prepared in the Cell Biology Core Lab, Institute of Plant and Microbial Biology, Academia Sinica. **Figure 1** was created with BioRender.com. The research data supporting this publication are provided within this paper. The authors thank both reviewers for their constructive suggestion to improve this manuscript.

## Conflict of interest

The authors declare that the research was conducted in the absence of any commercial or financial relationships that could be construed as a potential conflict of interest.

## Publisher’s note

All claims expressed in this article are solely those of the authors and do not necessarily represent those of their affiliated organizations, or those of the publisher, the editors and the reviewers. Any product that may be evaluated in this article, or claim that may be made by its manufacturer, is not guaranteed or endorsed by the publisher.
